# Applications and Advances of Magnetoelastic Sensors in Biomedical Engineering: A Review

**DOI:** 10.3390/ma12071135

**Published:** 2019-04-07

**Authors:** Limin Ren, Kun Yu, Yisong Tan

**Affiliations:** School of Mechanical Engineering, Northeast Electric Power University, Jilin 132012, China; renlimin@neepu.edu.cn (L.R.); 2201700350@neepu.edu.cn (K.Y.)

**Keywords:** magnetoelastic sensors (MES), biomedical applications and advances, wireless and passive characteristics

## Abstract

We present a comprehensive investigation into magnetoelastic sensors (MES) technology applied to biomedical engineering. This includes the working principles, detection methods, and application fields of MES technology. MES are made of amorphous metallic glass ribbons and are wireless and passive, meaning that it is convenient to monitor or measure the parameters related to biomedical engineering. MES are based on the inverse magnetoelastic (Villari) effect. When MES are subjected to mechanical stress, their magnetic susceptibility will change accordingly. And the susceptibility of MES is directly related to their magnetic permeability. The varying permeability can positively reflect the applied stress. The various detection methods that have been developed for different field applications include measurement of force, stress, and strain, monitoring of various chemical indexes, and consideration of different biomedical parameters such as the degradation rate and force conditions of artificial bone, as well as various physiological indexes including ammonia level, glucose concentration, bacteria growth, and blood coagulation.

## 1. Introduction

Magnetoelastic sensors (MES) have attracted considerable attention; as being wireless and passive, they can be used in biomedicine fields widely. Taking this into consideration, applied force [1], stress [2,3,4], pressure [5], and strain [6] can be determined using MES. By applying surface coatings which react with specific compounds, the mass of an MES can be changed, thus allowing remote monitoring of chemicals. Examples of parameters that have been monitored in this way are temperature, humidity [7], carbon dioxide [8], ammonia [9], pH [10], biological parameters such as glucose concentration [11], bacteria growth/presence [12,13] and coagulation [14], biliary stent monitoring systems [15], adhesion [16,17], to investigate the host response after an implant is embedded [18],and the biodegradation and biocompatibility of the MES [19]. MES can also be investigated by monitoring the change in their permeability with applied stress [20]. Examples of this include bone plate strain monitoring [21], the degradation rate of artificial bone [22,23], force monitoring of artificial bone [24], two-dimensional stress monitoring [25], and the tensile force on sutured wound sites [26].

MES are composed of amorphous ferromagnetic ribbons [27], most of which are iron-rich alloys such as Fe_40_Ni_38_Mo_4_B_18_ (Metglas® 2826 MB) [10,11]. Metglas 2826 MB is with a high mechanical tensile strength (~1000–1700 MPa) and a low usage cost, allowing them to be used cheaply. The magnetoelastic coupling factor of Metglas 2826 MB is 0.98 and the magnetostriction is 12 ppm [28,29]. Metallic glass ribbons of Metglas 2826 MB [30,31,32] are processed by single roller melt spinning technique. The Metglas 2826 MB is made by rapid heat quenching through the melt spin extrusion technique. The special operation results in a metallic strip ten microns thick, which makes the Metglas 2826 MB have excellent soft magnetic properties, such as high permeability, low coercivity, and hysteresis. The physical properties and magnetic properties of Metglas 2826 MB, the most popular MES material, are presented in Table 1. MES resonates when they experience an alternating magnetic field at their mechanical resonance frequencies. Depending on material selection, small MES resonates in the range of tens of KHz to several MHz.

Because a magnetic field is not significantly attenuated by human tissue when compared to an electric field, MES are more desirable for use in biomedical applications than inductive-capacitive sensors [33,34,35,36,37,38,39]. This is because they are wireless and passive. Particularly if the MES requires implantation in deep tissue, a wireless magnetic readout is advantageous.

The major advantage of MES is their wireless, passive (no battery) characteristics. MES are remotely detected by monitoring their magnetization at harmonic frequencies with a sensing coil under the excitation of an alternating magnetic field generated by an exciting coil [5,40,41,42]. Since there is no direct touch between MES and the detected object, MES can be applied for real-time and in vivo force condition tracking. In addition, MES do not need internal power to work, which makes further miniaturization available without compromising battery life. MES are able to detect all kinds of force conditions for their magnetoelastic property [43,44,45], which permits the magnetic permeability of MES to vary through an external applied force [3,46]. This is because the captured signals of MES are accordant with the magnetization of the sensor, and the magnetization of MES is directly related to the material’s magnetic permeability. Due to their small size, low cost, long lifetime, and passive and wireless characteristics, MES are ideal for biomedical applications.

## 2. MES Detection Theory and Method

### 2.1. Magnetic Materials Theory 

**Figure 1:**
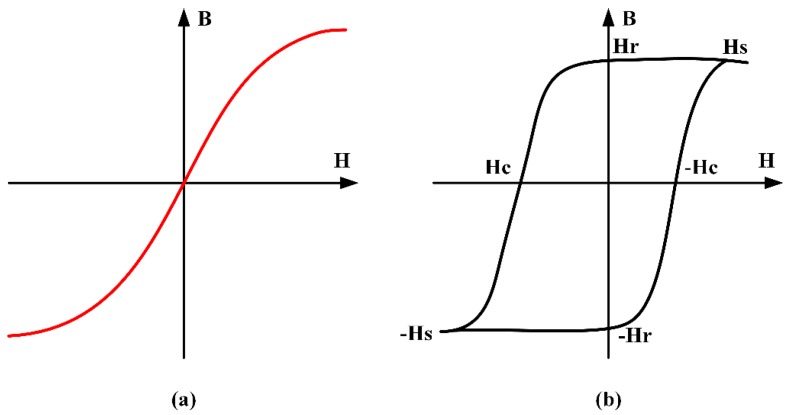
(**a**) *BH*-Loop of a soft magnetic material; (**b**) *BH*-Loop of a hard-magnetic material.

Soft magnetic material is a commonly utilized magnetic sensor [47,48,49]. Perfectly soft magnetic material is a material that magnetizes when exposed to a magnetic field, and demagnetizes completely when the field is removed. The concept can be visualized [50] by the *BH* loop shown in Figure 1a, which shows the magnetic flux density field *H*. In contrast, a hard-magnetic material [48,49,51] (Figure 1b) will magnetize up to a certain threshold (saturation magnetization *H_S_*) when exposed to an applied field, but will have some remaining field after the magnetic field is cancelled, called the remanence *H_r_* [52,53]. An additional magnetic field of opposite polarity would then be needed to demagnetize the magnetic material, which is referred to the coercive force *H_c_* [52,54,55,56,57]. The ideal magnetic material for use in most sensing applications would exhibit a minimum hysteresis, and thus, zero remanence and coercive force [58].

Magnetoelasticity refers to the phenomenon that the coupling of magnetic and elastic/mechanical energies in the material [59]. In other words, MES exhibit a mechanical deformation with an applied magnetic field, and a magnetic change when exposes to a mechanical stimulus. Specifically, a stress applied to MES will change their magnetic permeability *μ* [60], which is the slope of the linear portion of the *BH* characteristic. Conversely, an applied magnetic field will cause a variation in the magnetic material’s dimensions, as given by [61]:

(1) $$\varepsilon =\frac{\sigma }{E}+\frac{3\lambda_{s}}{2}\left(\frac{H^{2}}{H_{k}^{2}}-\frac{1}{3}\right),\;H<H_{k}$$

where *ɛ* is the induced strain, *σ* is the longitudinal stress, *E* is the Young’s modulus, *λ_s_* is the saturation magnetostriction, *H* is the applied magnetic field, and *H_k_* is the anisotropy field. The strain resulting from an applied magnetic field is known as the material’s magnetostriction, while the variation in the applied material’s Young’s modulus is known as the Δ*E* effect. The magnetostriction and Δ*E* effects occur due to rotation of the magnetic domains to align with the applied field direction, causing stretching along the direction of the field, and also inducing an internal strain on the material that causes the change in the material’s elasticity. 

In magnetoelastic materials, the reverse situation is true; stressing or straining a magnetic material can generate a variation in its preferred magnetization direction. These phenomena are called the Villari effect [62], or most often, stress-induced anisotropy *H_k_* [27]:

(2) $$H_{k}=\frac{2K_{u}-3\lambda_{s}\sigma }{M_{s}}$$

Their susceptibility *χ* [54] also changes, according to:

(3) $$\chi =\frac{M_{s}^{2}}{2K_{u}-3\lambda_{S}\sigma }$$

where *K_u_* is the uniaxial magnetic anisotropy energy coefficient, and *M_s_* is the magnetization.

The magnetic material’s susceptibility is directly correlated to the magnetic permeability of magnetic material [58]:

(4) $$\mu =\mu_{0}(1+\chi )$$

where *μ* is the permeability, and *μ*_0_ is a permeability constant.

The application of an external magnetic field causes the magnetic domains in the material to rotate along the direction of the field due to the Villari effect. This causes the materials to exhibit an elongation in the applied field’s direction. Upon excitation with a sinusoidal magnetic field, the material will exhibit a longitudinal vibration. At the mechanical resonant frequency, constructive acoustic wave energy will cause a maximum in vibration amplitude, thus causing the permeability of the material to rise. Since the acoustic wave propagation corresponds to the magnetic domain orientation, the sensor produces a secondary magnetic flux which exhibits a local maximum at mechanical resonance. This can be detected by a number of methods; most of which rely on detecting the voltage in the sensing coil originating from the induced voltage due to the time-varying magnetic field response from the MES, according to Faraday’s law of induction.

### 2.2. MES Detection Method

**Figure 2:**
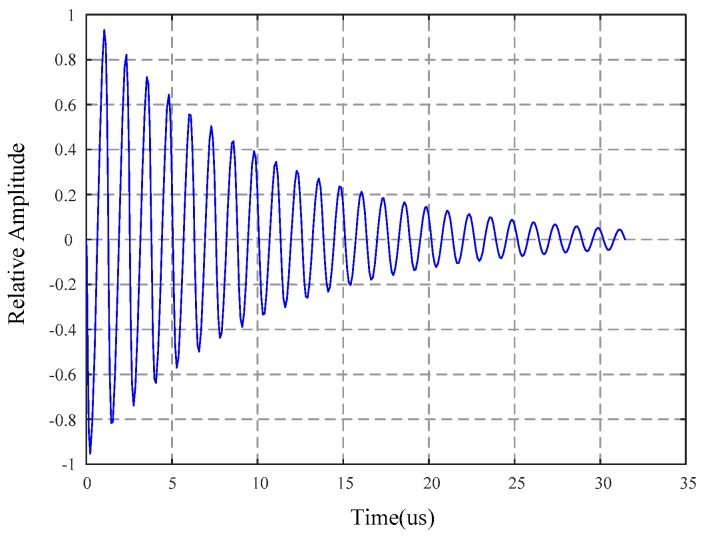
Typical shape of a ring-down response of a magnetoelastic sensor after the excitation field is removed.

The most common method for detection of the magnetostrictive effect of MES reported today is by monitoring the sensor resonance as a function of the parameter. Like any mechanical structure, a magnetoelastic sensor exhibits a mechanical resonance behavior depending on its mass, elasticity, and any damping forces applied. A typical rectangular resonant sensor will have resonant frequencies, as given by [63]:

(5) $$f_{n}=\frac{n}{2L}\sqrt{\frac{E}{\rho (1-v^{2})}}\;n=1,2,3\ldots \ldots$$

where *E* is the Young’s modulus, *ρ* is the density of the material, *L* is the length of the rectangular sample, *v* is the Poisson’s ratio, and *n* indicates that resonance occurs at integer multiples of the fundamental resonant frequency (*n* = 1), as shown in Figure 2. For Metglas 2826 MB, a commonly used magnetoelastic thick film, the resonant frequencies for MES from 1 to 100 mm resonate in the range of about 22 kHz (at 100 mm) to 2.2 MHz (at 1 mm). High precision magneto-quartz sensor also uses MES method to detect magnetostrictive effect, and adopts a new switching sensing principle, which has high sensitivity and temperature compensation ability [64,65].

One method for measuring the resonant frequency of an MES is by detecting the induced voltage in a detection solenoid, with the MES acting as the solenoid core. As the MES approaches resonance, the magnitude of the permeability changes, thus increasing the inductance of the coil *L*, given by:

(6) $$L=\frac{\mu N^{2}}{l}$$

where *μ* is the permeability of the core, *N* is the number of turns of the coil, and *l* is the length of the solenoid.

Frequency domain detection is also possible for monitoring the resonant frequency [66] of MES. One form of frequency detection used to monitor MES is to apply a known sinusoidal excitation and monitor the detection response at each frequency. Fourier transformations applied to the response are used to determine the detection amplitude as the applied sinusoidal field is stepped through a range of frequencies. The resulting frequency spectrums corresponding to each excitation frequency can be compared, where the highest amplitude will correspond to the resonant frequency.

The measurement method of the inverse magnetoelastic effect (Villari effect) [67,68,69,70,71] is focused on the material’s susceptibility; applied force can change the susceptibility, and the susceptibility can lead to a variation in the permeability. Meanwhile, the changing permeability of MES can cause a variation of spatial magnetic fields, which can be captured by the sensing coil.

**Figure 3:**
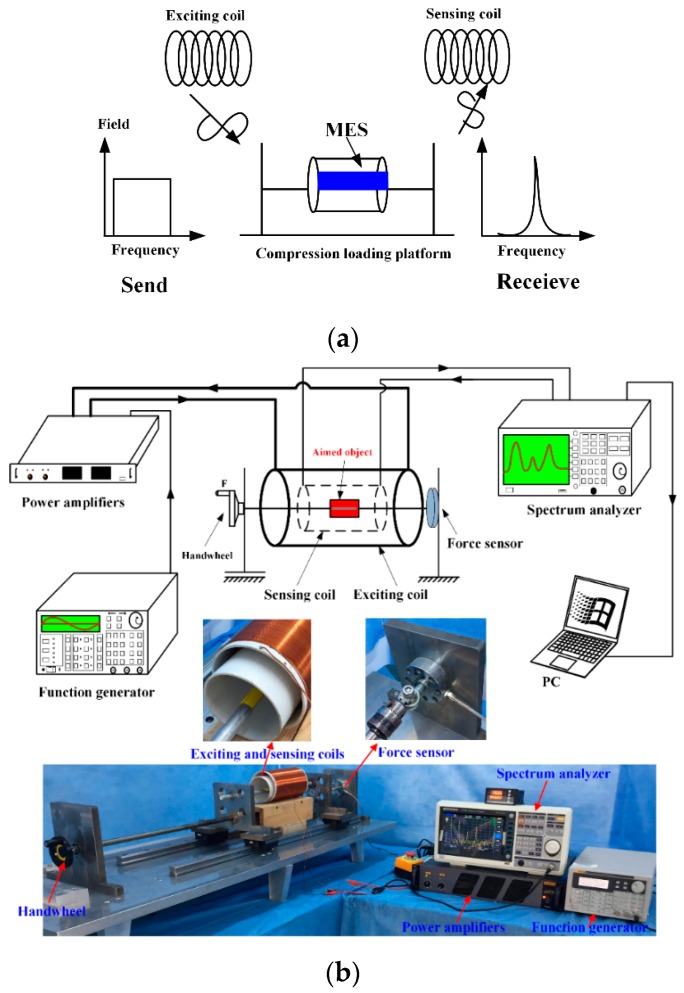
(**a**). Working principle and basic measure devices of the magnetoelastic sensor (MES). (**b**). Self-developed experimental platform and devices. Aimed object including MES is fixed on the platform and surrounded by the sensing coil and the exciting coil. Red color represents the aimed object including MES.

The working principle and basic measured devices of MES is shown in Figure 3a. There are two coils which are the exciting coil and sensing coil respectively. The dimension of the exciting coil is 110 mm, and the length is 200 cycles. The diameter of the copper wire used for the exciting coil is 0.5 mm. The dimension of the sensing coil is 90 mm, and the length is 200 cycles. The diameter of copper wire used for the sensing coil is 0.25 mm. The exciting coils generate an alternating magnetic field when being connected to an external sinusoidal source. MES suffer from a strain under the alternating magnetic field and generate the Villari effect (inverse magnetostrictive effect), which results in a magnetic flux variation induced by the sensing coil. The sensing coil, obtaining the changed signals, is linked with a spectrum analyzer. The collected data are reserved in a desktop computer. 

MES are used to measure strain [21]/stress [25]/degradation rate [22,23]/force condition [24]. MES are completely integrated with aimed objects by healthy adhesive such as artificial bone/elastomer/bone plate. Then we use the self-developed platform and equipment to measure the varying conditions of MES. The experimental platform and equipment are shown in Figure 3b.

The aimed object including MES are fixed by the force-loading platform and placed inside of the copper coils that are exciting coil and the sensing coil. The handwheel is used to apply the external force on the aimed object, which causes certain stress working on the MES and a change in the magnetic permeability of MES. The variation of the magnetic permeability can cause a change in the external applied magnetic field, that is detected by the sensing coil through a wireless and passive method. The increase of the permeability is accordant with the applied stress in a certain scope.

## 3. Biomedical Applications of MES

### 3.1. Bone Plate Strain Monitoring

**Figure 4:**
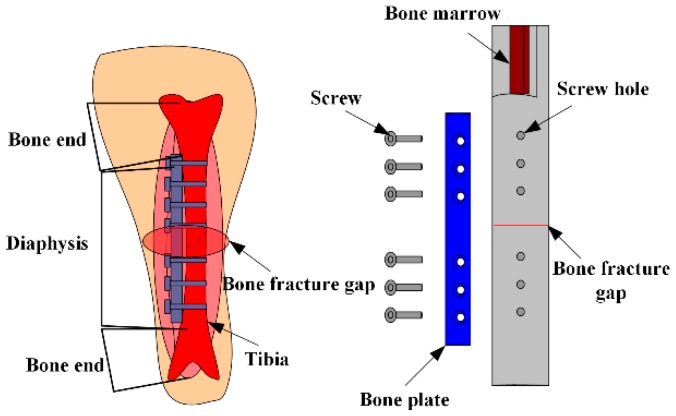
The repair model of sheep tibia.

**Figure 5:**
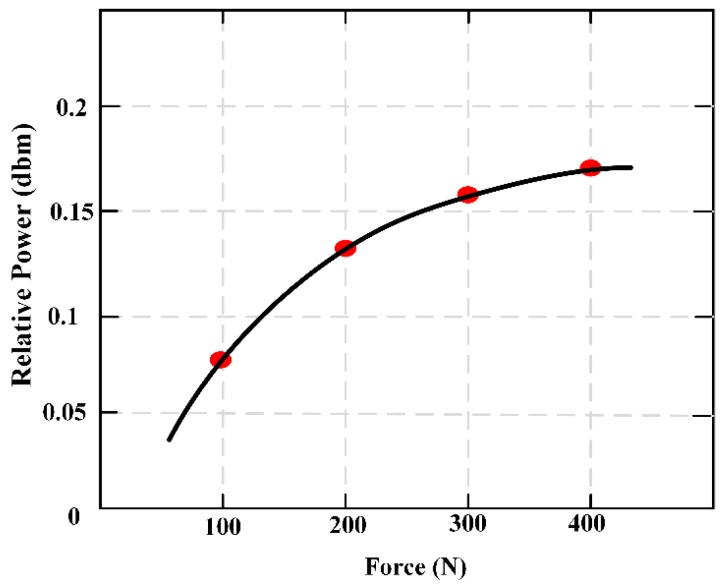
The external applied force (F) and relative powers (dbm).

An MES used for monitoring bone plate strain in real-time, and for judging the healing state of fractures in patients, was proposed [21]. A tibia-bone plate-screw (TBS) model, shown in Figure 4, was designed using the finite element analysis model, and a sheep tibia was chosen as the model of bone fracture. Measurement points out that the strain of bone plate decreases with the bone slowly healing, the result is consistent with the finite element analysis. This verifies the reliability of the sensors presented here. The results can be used not only in the clinical application of fracture healing, but also in the design of fracture treatment and rehabilitation equipment. Figure 5 maintains the relationship of the external applied force (F) and relative power (dbm).

### 3.2. Force Monitoring of Artificial Bone

**Figure 6:**
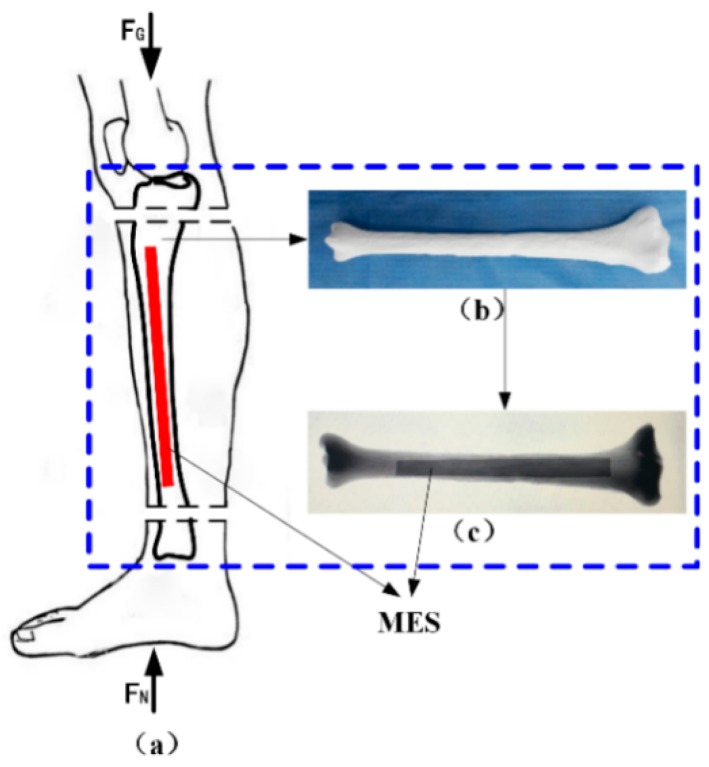
Application of the AB (artificial bone). (**a**) Human shank model with AB, (**b**) 3D printing AB and (**c**) X-ray picture.

**Figure 7:**
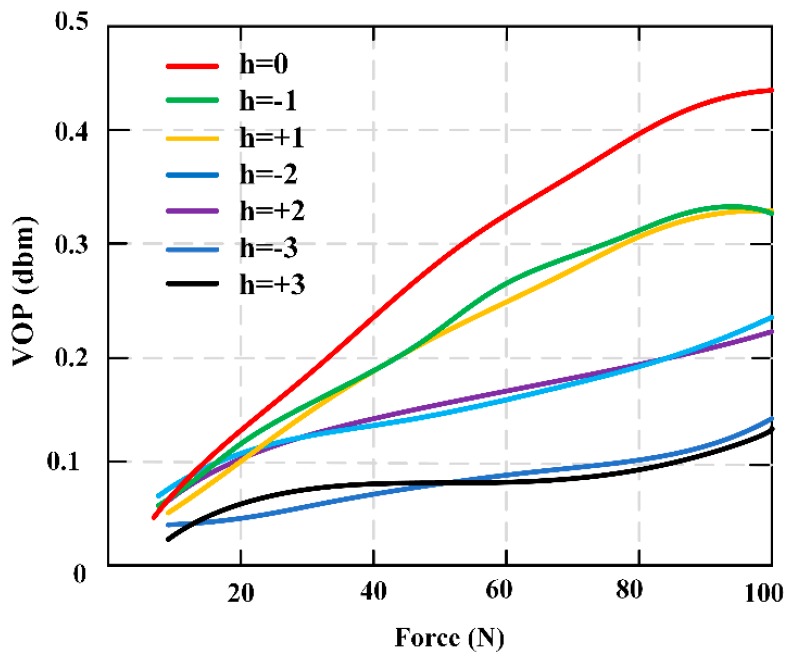
The varying values of output power (VOP) and the force condition of the artificial bone (AB) embedded magnetoelastic sensor (MES) in seven different positions.

A novel monitoring method for mechanical properties of artificial bone (AB) based on inverse magnetoelastic effect (Villard effect) was established. A novel 3D printing technology was used to embed the MES inside AB. Seven AB models were proposed, designed, fabricated and measured using self-developed experimental equipment. The optimal output position of MES was selected. The relationship of external applied force (*F*) and relative power (dbm) was discussed. MES was used to further monitor the performance of AB covered by certain area biological tissues. The applications of MES and AB are shown in Figure 6. The results show that the middle position of AB (h = 0) can completely demonstrate the performance of MES, and the force performance of AB can be clearly expressed and monitored by wireless and passive methods. This further indicates that MES can effectively monitor the mechanical properties of AB and prosthesis in vitro and in vivo. Figure 7 shows the varying values of output power (VOP) of the MES at different positions of AB, with the external force increasing. Importantly, the MES is first implanted in the middle position of the AB to explore the force condition, which can eliminate all kinds of interference and directly reflect the real conditions through a wireless and passive method.

### 3.3. Degradation Ratio of Artificial Bone Made of Magnesium Alloy

**Figure 8:**
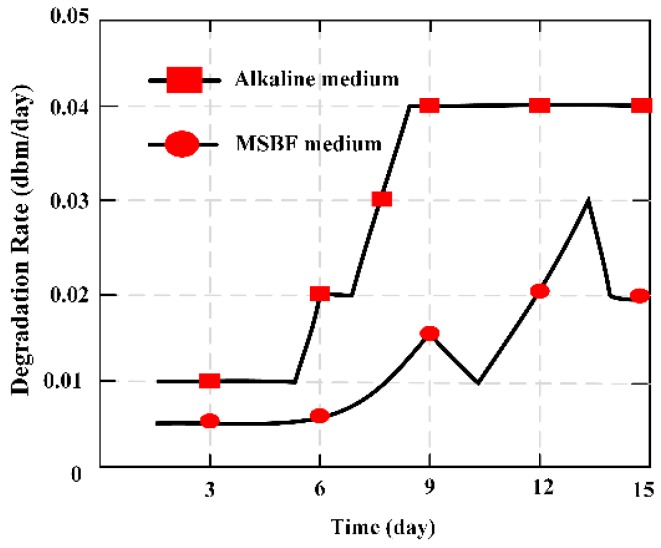
Degradation ratio of the MES (magnetoelastic sensors)-embedded MBAB (magnesium-based artificial bone) EMBAB (MES-embedded MBAB,) at increased time points in alkaline and MSBF (modified simulated body fluid) media over 15 days.

MES was applied as a novel measuring method in vitro to evaluate the degradation ratio of magnesium-based artificial bone (MBAB), which could be applied as an artificial implant to repair or treat a bone defect and fracture. It offers a quantitative way to illustrate the degradation ratio of the MBAB [23]. The MES was implanted in the middle position surface of MBAB using a non-harmful quick adhesive, and 3D printer forming of the MES-embedded MBAB (EMBAB). A gradually increasing strain could directly work at the MES with the EMBAB’s degradation, significantly varying the value of the relative output power. The degradation ratio of the EMBAB can be calculated on the basis of the variation of the relative output power caused by the MES, and the degradation time given by Equation (7). The MES presents a novel method to evaluate the degradation ratio of bone substitutions in vitro. Figure 8 indicates the degradation ratio of the EMBAB.

(7) $$Y(\mathrm{dbm}/\mathrm{day})=\frac{Q_{n}}{T_{n}}$$

where *Y* is the degradation rate of an EMBAB, *T_n_* is the time at which the degradation is measured (*T_n_* = 1, 2, …, 15), and *Q_n_* is the varying value of the relative output power at day *n*.

To evaluate the degradation ratio of artificial bone made of magnesium alloys, two common methods [40] were usually adopted, namely the weight loss method [41] and the hydrogen evolution method [42]. An MBS was employed firstly to evaluate the degradation ratio of MBAB. Hence, the common method of weight loss was used to evaluate the degradation ratio of MBAB and further confirm the ability of the MBS. The calculated equation is given:

(8) $$X(g/\mathrm{day})=\frac{M_{n}}{T_{n}}$$

where *M_n_* is the varying value of the quality of MBAB at day *n*, and *T_n_* = 1, 2, …,15 is the time between two measurement instants.

**Figure 9:**
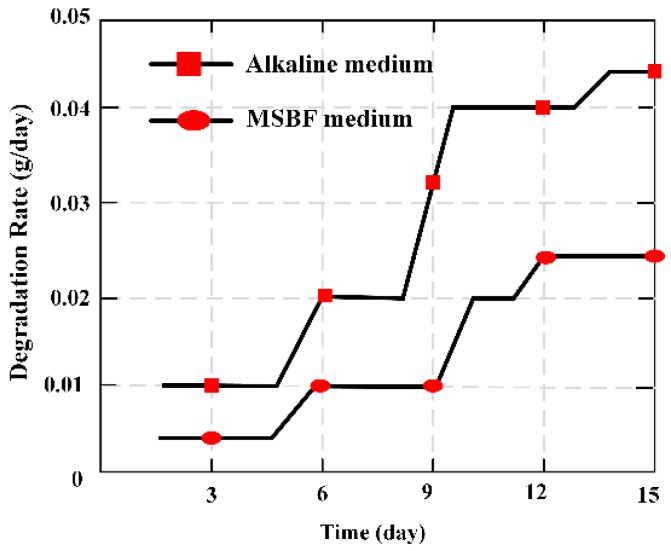
Degradation ratio (g/day) of MBAB with adding degradation time in alkaline medium and modified simulated body fluid (MSBF)medium over 15 days.

The varying trend of degradation ratio *X* (g/day) was calculated by the familiar method of weight loss indicated in Figure 9, and was similar with the *Y* (dbm/day) that figured out through the output value of relative power of the MES. Hence, the relative output power of the MES can be applied to evaluate and illustrate the condition of degradation ratio of MBAB.

The magnetism plate was coated on the both sides of the MES, forming the MBAB, and the MES was used to evaluate and demonstrate the degradation ratio of MBAB in alkaline medium and modified simulated body fluid (MSBF) medium. It is an ideal measurement to evaluate the degradation condition of degradable materials through a wireless and passive method, making it a useful measurement technique for biomedical engineering.

### 3.4. Two-Dimensional Stress Monitoring

**Figure 10:**
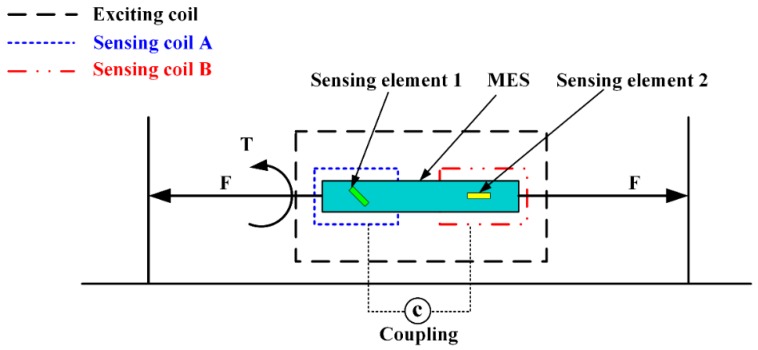
The working principle of two-dimensional stress on magnetoelastic sensor (MES) using the Villari effect.

**Figure 11:**
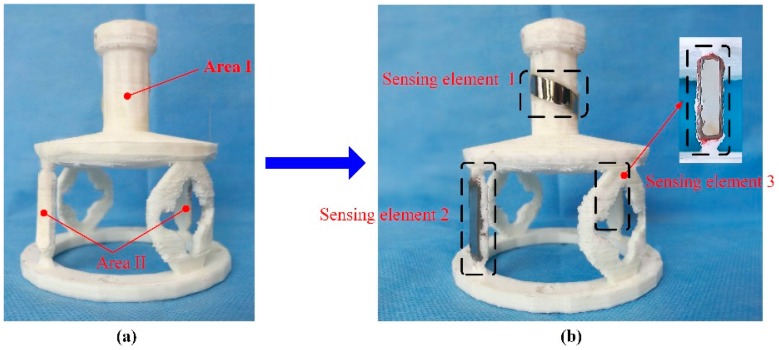
The 3D entity model structures of the designed sensor: (**a**) the elastomer structure model and (**b**) the pasted positions of the three MESs.

**Figure 12:**
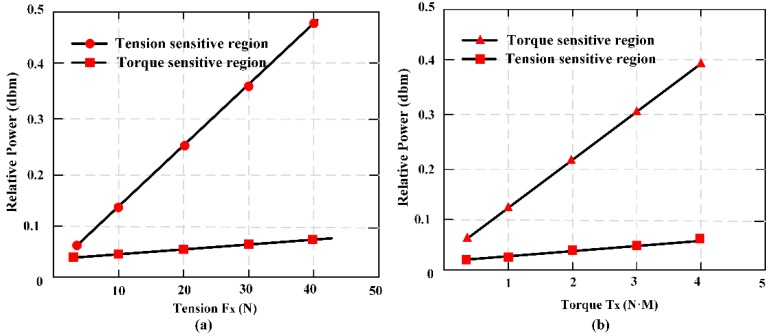
The condition of output performance and sensor’s linear fitting condition: (**a**) output condition of tension and (**b**) output condition of torsion torque.

A novel two-dimensional stress sensor with the characteristics of wireless and passive employing the Villari effect (inverse magnetostrictive effect) was proposed and designed [25]. Three pieces of MES were fully pasted on the easy deformation zones of an elastomer structure to establish the sensor. The effective measurement ranges of the sensor are 0–40 N in tension and 0–4 N·M in torque respectively. The sensor error of *F_x_* is 3.4%, and of *T_x_* is 4.2%, thought test load (35 N and 3.5 N·M). The passive and wireless sensor is applied for a long-term detection of mechanical loading within a moving object. Figure 10 shows the working principle of MES applied to two-dimensional stress monitoring. Figure 11 presents the 3D entity model structure of the designed sensor: (a) The designed elastomer and (b) the pasted positions of the three MESs. Figure 12 shows the condition of output performance and sensor’s linear fitting condition: (a) Output condition of tension and (b) output condition of torsion torque.

The applied load can be represented by the force matrix of *F*, and the output data can be expressed in the matrix of *U*. Then, the quantization relationship between the input matrix and the output value matrix can be expressed as follows:

(9) $$F=\left[\begin{array}{cc} F_{x} & 0\\ 0 & T_{x} \end{array}\right]U=\left[\begin{array}{cc} U_{11} & U_{12}\\ U_{21} & U_{22} \end{array}\right]F=\left[\begin{array}{cc} C_{11} & C_{12}\\ C_{21} & C_{22} \end{array}\right]\cdot U$$

It can be abbreviated as follows:

(10) F=C×U

where *C* is the calibration matrix, which is also called the coupling matrix because it can reflect the coupling relationship between the forces of each of the dimensions. Hence, the coupling matrix, *C*, could be obtained using Equation (11). The function relation is as follows:

(11) $$C=F\times U^{-1}$$

*C* is obtained and shown in Equation (12), as follows:

(12) $$C=\left[\begin{array}{cc} 79.0960 & -14.8305\\ -0.9887 & 11.1229 \end{array}\right]$$

From the calibration matrix, *C*, it can be concluded that the coupling component is very small. In the second row of the matrix, the proportion of the coupling component and the main component is 1:11.2 (0.9887/11.1229). This indicates that the structure proposed in the paper has a certain ability for self-decoupling. 

In order to obtain the actual performance of the sensor, a composite force loading experiment was carried out on the sensor. A compound test force (35 N) and torque (3.5 N·M) were applied to the sensor at the same time, and the output data from the two sensing coils were obtained. Then, by using the calibration matrix, *C*, and the sensor output data, *U*_0_, which were collected in the composite force loading experiment, the two-dimensional force matrix, *F*_0_, could be obtained by introducing it into Equation (10).

(13) $$U^{\prime }=\left[\begin{array}{cc} 0.46 & 0\\ 0 & 0.33 \end{array}\right]F^{\prime }=\left[\begin{array}{cc} 36.3842 & -5.1907\\ -0.4548 & 3.6706 \end{array}\right]$$

(14) $$\Delta =\left|\frac{F^{\prime }-F}{F_{\max}}\right|\times 100\%$$

After the linear calibration decoupling, we compared the results obtained to the *F*’ = (36.3842, 3.6706) and the input force matrix *F* = (35, 3.5); the errors of the two-dimensional wireless passive sensor could be calculated using Equation (14). Finally, the errors of the sensor were obtained as follows: *F_x_* is 3.4% and *T_x_* is 4.2%.

A two-dimensional stress monitoring sensor was proposed based on MES and the elastic structure. The tensile and torque force can be measured simultaneously by the sensor. It is indicated that the structure proposed in this paper has a certain ability for self-decoupling, which means that it is very easy to monitor the two-dimensional force using the smart structure.

### 3.5. Tracking Degradation Profiles of Nitro Dopamine-Modified Poly

**Figure 13:**
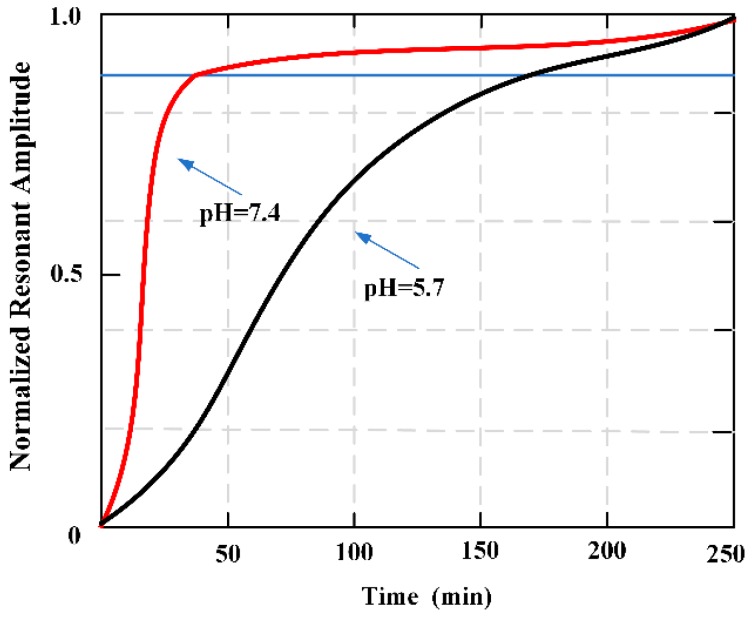
Change in the normalized resonant amplitudes of magnetoelastic sensor (MES) when coated hydrogel degraded them at pH 5.7 and 7.4. The curves represent the average value of three samples.

Fast degrading, biomimetic PEG-(Glu-ND)_4_ was pasted on the applied MES strips, and the degradation behavior about the adhesive used was monitored though tracking the resonant amplitude of the sensor [72]. The resonant amplitude increased overtime, corresponding to the mass loss of the adhesive. Additionally, degradation behavior observed using MES matched the qualitative degradation of the bulk adhesive. Oscillatory rheometric was used to confirm the formation and degradation of PEG-(Glu-ND)_4_. This sensing technology is demonstrated as a potentially useful tool for evaluating the degradation rate of bioadhesives. Figure 13 plots the change of the resonant amplitude for the adhesive-coated sensors as they degraded in pH 7.4 and 5.7.

### 3.6. MES Array System Controlling Cell Adhesion

**Figure 14:**
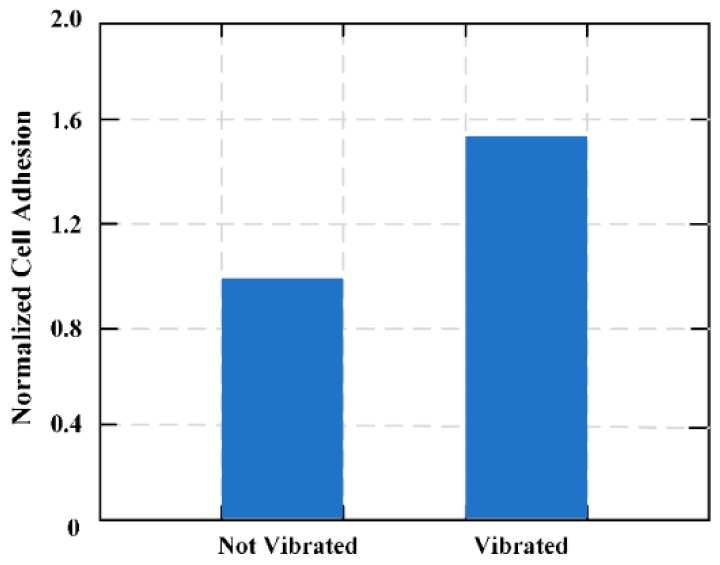
Effects of vibrations (<10 nm) on fibroblast adhesion to plasma-treated Paryene-C coated magnetoelastic sensor (MES) films.

A novel system was proposed and designed to selectively control and command cellular adhesion to biomedical implants. The system was based on remotely configurable MES to produce submicron mechanical vibrations at predetermined amplitudes and frequencies [18]. An MES membrane composed of two connecting bands was designed and the regional control ability of the system for cell adhesion was calculated. In vitro cell culture experiments with L929 fibroblasts shows that by changing the frequency of the magnetic field, cell adhesion can be increased or decreased in different areas of the membrane. Figure 14 shows the effects of vibrations on the adhesion of plasma-treated Parylene-C coating MES films.

The MES material was designed to selectively regulate cell adhesion at the tissue-biomaterial interface. This is the first biomaterial method to date that uses submicron mechanical loads to control the material-surface-tissue interaction. The noninvasive activation of the coating contrasts with current surface modification techniques that can be adjusted in situ depending on the life of the implant. The coating can be externally vibrated after implantation to control cell adhesion, and the surface conditions can be quantified in real-time by induction of a secondary magnetic field. This secondary reaction will provide a unique capability for real-time monitoring of biological reactions on implant surfaces. Combined with micro and nanoscale surface modification, this method may introduce “self-perception and post-deployment activation” components in implant design to alleviate host response and reduce the need for surgical modification.

### 3.7. Tensile Force on Sutured Wound Sites

**Figure 15:**
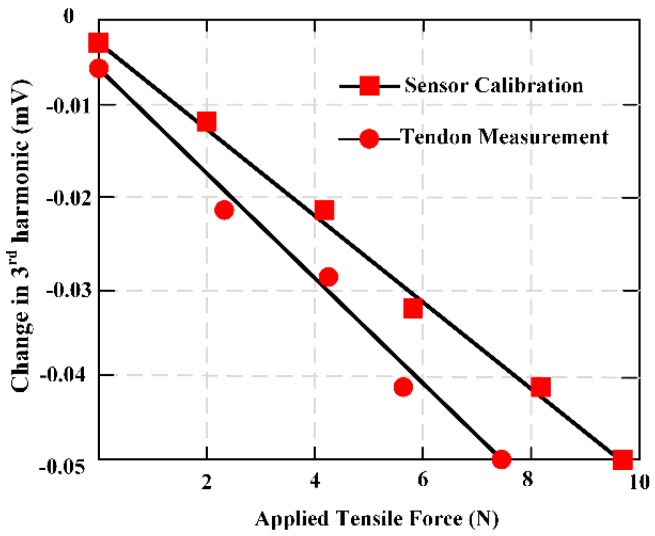
The force measurement through the magnetoelastic sensor (MES), the tendon was loaded from 0 to 10 N. MES calibration measurements are also shown in the red circles.

A novel wireless MES was developed and applied to the field monitoring of wound tension. Both ends of MES were attached to a suture line to secure the sensor at the wound repair site [26]. This work proposed two sensor designs and fabrication: One for high ranges and another for low force ranges. A sensor was fabricated by directly pasting the MES on the suture. This sensor demonstrates good sensitivity under low force, but its response was saturated at about 1.5 N. To evaluate and illustrate high tensile force, the MES was pasted on another one metal strip to share the load. The sutures were attached to both ends of the metal strip so that only a small part of the force was applied directly to the MES, showing good sensitivity even at 44.5 N. The results indicated the potential for in vivo force monitoring of physical activity of wound repair site. Figure 15 shows the third harmonic amplitudes of the MES is obtained when the deer tendon is subjected to a force of 0 to 12 N.

In order to ensure clinical practicality, the MES needs to be much smaller than its current size. MES can be miniaturized by changing manufacturing process. For instance, shearing a shorter, thinner sensor and attaching it to a thinner backing material may produce a smaller sensor with the same design process. In addition, change in manufacturing techniques, such as electroplating or sputtering of existing sutures, may allow for the manufacture of smaller sensors. 

Nevertheless, smaller sensors lead to a lower signal amplitude, so more sensitive sensing coil and amplifier will be applied. MES can also be added to existing sutures, so closed wound sutures can also be used as sensing sutures. To achieve this, a biodegradable, non-cytotoxic magnetoelastic material [19] could be functionalized onto the suture. Because the material is bioabsorbable and is applied in the biodegradable sutures, it is not necessary to dismantle the sensor after use.

### 3.8. Monitoring Coagulation, Clot Inhibition, and Fibrinolysis

**Figure 16:**
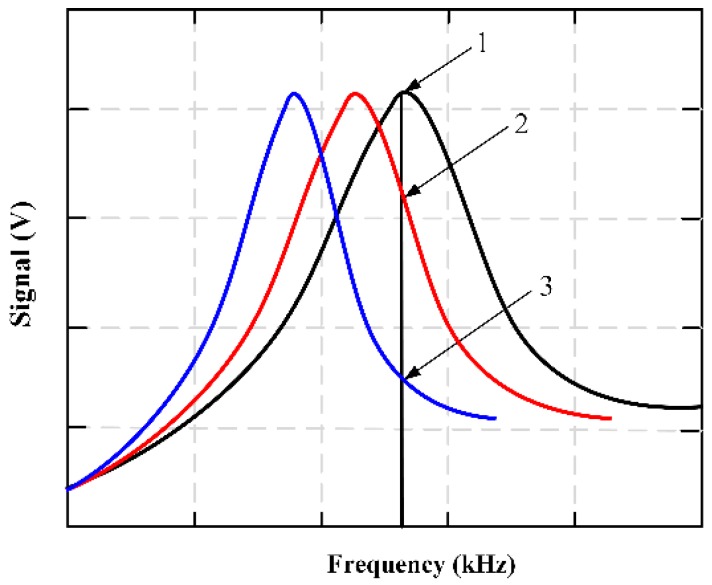
A graphical representation of the output signal obtained during the process of coagulation monitoring.

MES had been applied to detect and monitor the viscosity changes during the coagulation and fibrinolysis biological reactions [73]. The characteristic resonance frequency of the MES varies with the change of fluid viscosity. At a set frequency, the output signal can be obtained over time to form coagulation and/or dissolution profile showing changes in plasma sample viscosity experienced coagulation or fibrinolysis. Figure 16 shows how the output signal is obtained during coagulation monitoring. Before the solidification began, the set frequency was selected as the resonance frequency of the magnetoelastic strip containing the test mixture and marked with a vertical line. As the solution viscosity increases, the strip resonance frequency decreases (as shown by the red and blue curves).

**Figure 17:**
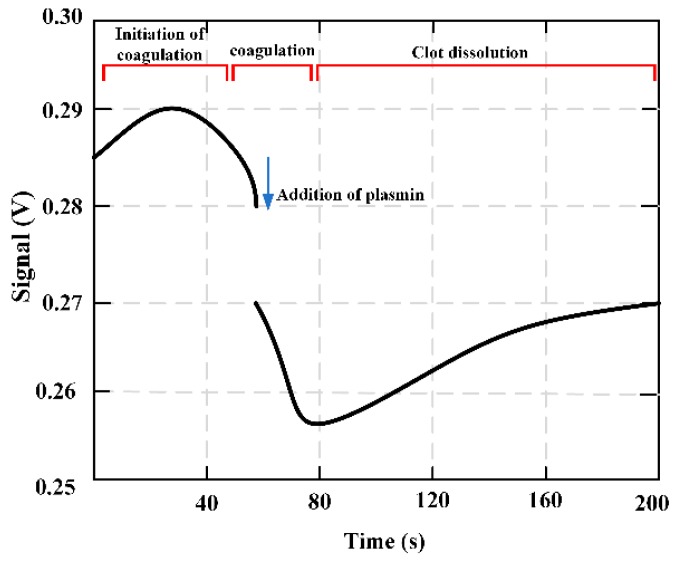
The fibrinolysis of the partially formed fibrin clot, and with the addition of plasmin, the viscosity still increases for a few seconds.

**Figure 18:**
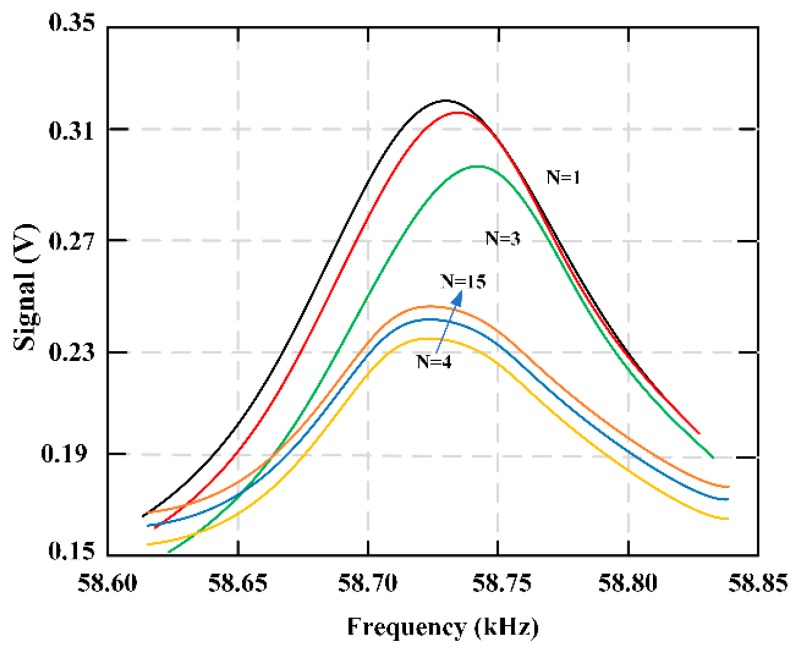
Multiple frequency sweeps of the fibrinolysis process illustrating the frequency change over time. Fifteen total scans were taken, with the addition of plasmin coming between the third and fourth scans.

Figure 17 presents the fibrinolysis of the partially formed fibrin clot. With the addition of plasmin, the viscosity still increased for a few seconds (indicating a decrease in signal). The third marker feature was an increase in signal, which pretended a decrease in solution viscosity. The variation of this signal is due to thrombolysis caused by the addition of plasmin. The qualitative feature of the marked area is the signal response to the change of solution viscosity. Figure 18 shows the multiple sweeping frequencies of the fibrinolysis process, indicating the change of frequencies with time. A total of 15 scans were performed, and fibrinolytic enzymes were added between the third and fourth scans. The delay time between each scan is about 36 seconds.

### 3.9. Mapping System for Biomedical Applications

**Figure 19:**
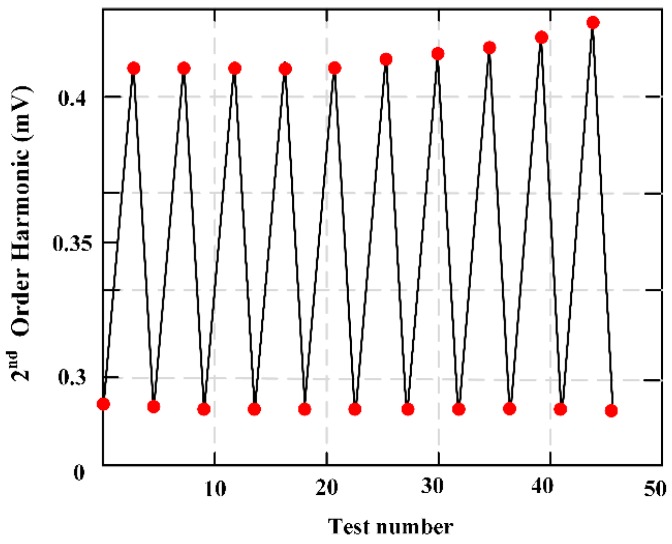
In the process of 10 loading cycles, the cyclic loading of the sensor ranged from 0.044 to 0.133 kN, and the results show the response drift of the sensor is very small.

A wireless, passive force-mapping system based on soft and amorphous MES permeability variation was presented for long-term force/stress monitoring of biomedical equipment [1]. Though real-time monitoring of the force distribution of the prosthesis interface, it is proved that this technology can be applied to lower limb prosthesis to ensure correct postoperative cooperation. Figure 19 expresses the MES cyclic loading executed form 0.044–0.133 kN in 10 loading cycles, and results show that the MES response has a low drift.

The sensor system consisted of a force-sensitive MES array that monitored and demonstrated the applied load and as the observation of the variation in the peak amplitude of the magnetic high-order harmonic signal measured by each array element. The change of high-order harmonic signal is caused by the variation of the permeability of MES, which corresponds to the increase of MES magnetization. After loading, the measured high-order harmonic signals were input into the algorithm to decide the external applied force, so as to determine the real-time loading contour at the junction of bodywork protection parts.

## 4. Considerations and Perspectives

Metglas 2826 MB ribbon is popularly used in biomedical engineering, and is made of Fe_40_Ni_38_Mo_4_B_18_. However, the Ni and Mo elements of the MES are toxic. They cannot directly touch human tissue and organs. This type of MES is coated fully with health quick adhesive to a thickness of about 10 μm, and then the MES is embedded completely in the neutral surface of magnesium-based artificial bone (MBAB). When the MBAB in the human body has fully degraded, a micro-operation is used to remove the MES. It is also possible to use MES in vitro to measure and monitor blood coagulation [14], and the host response (wound healing) to an implant in vivo [18]. The MES is applied to monitor the tensile force on sutured wound sites [26], and for example, it was implanted in a rat’s 6 mm femoral segmental defect model to noninvasively measure and assess the strain in real-time [74]. This research demonstrates that toxic elements did not have any clearly negative effect on healthy individuals, however it is important for us to take measures to prevent the MES from directly touching tissue or organs. When using some protective measures, the magnetic alloy is suitable for implantation in a human being.

The change of temperature can cause the variation of the magnetoelastic coefficient, which increases with the change of the temperature. The temperature can determine the accuracy of applications to a certain extent. Therefore, the influence of temperature on MES should be carefully considered when used. The MES is tested at constant temperature of about 20 °C, and the strain field of different magnetoelastic materials varies nonlinearly, and the magnetoelastic coefficient increases rapidly with the increase of temperature from low temperature to room temperature (about 20 °C.). The magnetoelastic values of magnetoelastic materials are larger when the temperature is 40–50 °C. With the increase of temperature, the magnetoelastic value begins to decrease slowly [75]. After long uninterrupted loading and unloading, the temperature of MES will change. Therefore, MES should be tested every 3 hours in our own lab in order to reduce the influence of temperature on the experimental data and result.

The distance between MES and the exciting and sensing coil can directly affect the sensitivity of the signal. The exciting coil we used is 110 mm in diameter and 200 mm in length, which can produce an alternating magnetic field (3 × 10^2^ A/m) caused by a sinusoidal signal excitation (200 Hz and 2 V, peak to peak) and amplified by a power amplifier. And the sensing coil is 90 mm in diameter and 100 mm in length. The aimed object including the MES is always surrounded with the two coils, the maximum distance between the MES to exciting coil (outer coil) is 55 mm and to the sensing coil (inner coil) is 45 mm. MES is a continuous ribbon of 11.7 mm in width and 28 μm in thickness [28], and the length depends on our aim object. The magnetoelastic coupling factor is 0.98 and the magnetostriction is 12 × 10^−6^ [29]. In our experimental condition, the sensing coil can accurately reflect the sensitivity of the signal for the distance between MES and the two coils is relatively small, which is only 55 mm in maximum. There are no other signals disturbing the experimental data around our lab. Of course, if the distance between MES and the two coils are relatively larger or the aimed object is not surrounded by the two coils, the distance will affect the sensitivity of signal. And if the aimed object is placed beyond the scope of the spatial magnetic field, the sensitivity of signal will also be affected. Hence, it is important to take care of the distance between MES and the sensing coil and the exciting coil, which can affect the sensitivity of the signal.

MES can work normally if they are surrounded with liquid in a live organism environment. Vlaisavljevich E. et al. [17] described the real-time evaluation and demonstrated capabilities of MES in the process of wound healing of mice. The in vivo study demonstrates that MES can monitor the implant in mice and control the local cell density and collagen matrix generated at the interface of soft tissue implant. The MES is placed in the wound surface to monitor the wound healing of mice, and it can clearly reflect the condition. MES have been used to evaluate and illustrate the viscosity variations that produce during the biological reactions of coagulation and fibrinolysis [14]. The results show that MES can work normally in a liquid environment. In other words, the MES is made of some toxic elements, such as Ni and Mo. Hence, some protective measures should be taken when MES is used in vivo.

MES are applied in biomedical engineering for their wireless and passive characteristics. Most of them are implanted inside the body, or indirectly touch tissue or organs. They provide a convenient way to measure/monitor all kinds of parameters, but further development of MES is necessary. As an implantable sensor, the degradable characteristic is a key point for MES, as it can reduce cost, pain, and the need for a second operation. The wireless and passive characteristics of MES, plus the degradable performance, can further improve and enhance their applications in biomedical field. The time scale of MES degradation should be carefully considered for it also means degradation of measurement accuracy. If the MES begins to degrade, the measuring function will gradually reduce until it is fully loss its function. MES should still work until the measurement is finished, but the condition is that its function will lose when beginning to degrade. Some special considerations should be taken to protect the function of MES. For instance, a thin film is pasted on the surface to delay the degradation. But the method also affects the sensitivity. In order to fully guarantee the accuracy of MES we should better redesign certain biodegradable magnetoelastic material in the future in order that the degradation characteristic should have no effect on the measuring characteristic. Only in this way, can the time scale not affect the measure accuracy when the magnetics materials degrading. The best condition is that the degradation and the measurement are synchronous, and each of them are not disturbed, then there is no necessary for us to care about the time scale. Hence, the development of optimal magnetoelastic materials is indeed needed. As for Metglas 2826 MB (the most MES material), the degradation process can affect the measure accuracy in some conditions.

Two-dimensional wireless and passive sensors are proposed firstly for monitoring the stress of tensile and torque based on the elastomer structure. Three pieces of MES are bonded on an elastomer smart structure base to form the sensor. The smart structure of the sensor is based on the stiffness distribution, which causes the sensor to have a particular decoupling ability. It can potentially be implanted in vivo to monitor the force information of bones and joints, in order to prevent failure due to overloading. Furthermore, it has the ability to detect the two-dimensional sensor force information at the same time, which is significant for the mechanical detection of orthopedic implants. Future work will focus on the improvement of the miniaturization of the structure, and its application in implantable biomedical sensors.

## 5. Conclusions

We presented here a comprehensive investigation of magnetoelastic sensors (MES), including operational theories, detection methods, and application fields in biomedical engineering. Magnetoelastic sensors are amorphous ferromagnetic ribbons that show a magneto-mechanical resonance when it is excited by a varying external magnetic field. An applied stress working on an MES can change its anisotropy and susceptibility, which are directly related to its magnetic permeability. The varying magnetic permeability shifts in response to different biomedical parameters, including stress, pressure, temperature, blood coagulation, clot inhibition, and fibrinolysis, and even the degradation rate and force conditions of artificial bone used to repair bone defects.

In order to understand the behavior of MES under different operating conditions, a variety of detection methods have been developed. The popular method for MES uses a function generator, power amplifiers, a spectrum analyzer, a sensing coil, an exciting coil, and a force loading platform. The function generator produces a sinusoidal signal amplified by the power amplifier, which is then input into the exciting coil to produce an alternating magnetic field. By applying an external force on MES through the loading platform, the MES is subjected to stress and the magnetic response field changes. The variation of MES will lead to the change of external magnetic field, which can be detected by sensing with a wireless and passive method. Importantly, under certain conditions, permeability increases with the rise of applied stress. The relationship between the external force on MES and the output power of the sensing coil is obtained. The applied force of MES is indirectly explained by the relative power. In this process, MES does not require cables and batteries, which means it is an ideal candidate for long-term implantation to evaluate and measure the condition of in vivo and in vitro force performance.

MES have attracted considerable interest in relation to biomedical engineering, because they form an excellent sensor platform that can be used to measure a wide range of environment parameters, including carbon dioxide, ammonia, pH, biological parameters, glucose concentration, bacteria growth/presence, coagulation, tensile force on sutured wound sites, biliary stent monitoring systems, adhesion, and to evaluate and illustrate the host response to an implant, as well as the biodegradation and biocompatibility of the MES. MES can also be interrogated by monitoring the change in their permeability with applied stress, such as in bone plate strain monitoring, the degradation ratio of magnesium-based artificial bone, force monitoring of artificial bone, and two-dimensional stress monitoring.

The next step for MES, from the development aspect of biomedical application engineering, should focus on the biosensor’s degradable characteristic. MES as implantable sensors have been used to monitor or measure all kinds of biomedical parameters in vitro and in vivo. The wireless and passive merits of MES could allow a new method of non-invasive monitoring for human. If MES were implanted in vivo, a second operation is currently required to remove it from the body after use. This not only increases the patient’s pain and cost, but also increases the treatment difficulty for doctors. Therefore, the exploration and study of degradable magnetoelastic biosensors should be prioritized in the future. Another future development for MES is the creation of multi-dimensional sensors for monitoring the multi-force working conditions in vivo or in vitro, based on different elastomer structures that are fabricated by the different biodegradable or biocompatible materials.

## Figures and Tables

**Table 1:** The magnetic properties and physical properties of Metglas 2826 MB, the most MES (Magnetoelastic sensors) material.

Magnetic Properties		Physical Properties	
Saturation Induction (T)	0.88	Density (g/cm^3^)	7.90
Maximum D.C. Permeability (µ):		Vicker’s Hardness (50 g load)	740
Annealed	800,000	Elastic Modulus (GPa)	100–110
As Cast	>50,000	Tensile Strength (GPa)	1–2
Saturation Magnetostriction (ppm)	12	Lamination Factor (%)	>75
Electrical Resistivity (µΩ × cm)	138	Continuous service Temp. (°C)	125
Curie Temperature (°C)	353	Thermal Expansion (ppm/°C)	11.7
Anisotropy field (A/m)	300	Crystallization Temperature (°C) Young’s modulus (GPa)	410 200
